# Usability and Feasibility of an In-Home Full-Color Imaging Scale for Remote Monitoring of High-Risk Patients With a History of Diabetic Foot Ulcers: Retrospective Observational Formative Evaluation

**DOI:** 10.2196/94016

**Published:** 2026-07-23

**Authors:** Emily Hu, Anuj Khandelwal, Eric Dahlseng, Navleen Saini, Dawn Gretz, Justin Lewis, Richard Silverstein, Nyanika Arora, Quincy Huynh, Bryan Markinson

**Affiliations:** 1 Empo Health, 881 Sneath Ln, Suite 111, San Bruno, CA, 94066, United States; 2 Maryland Foot and Ankle Specialists, Eldersburg, MD, United States; 3 Union Foot Care, 508 Lafayette Street, Havre de Grace, MD, 21078, United States, 1 410-939-2627, 1 410-939-4681; 4 Department of Orthopedics, Denver Health Hospital and Authority, Denver, CO, United States; 5 Icahn School of Medicine at Mount Sinai, New York, NY, United States

**Keywords:** digital health, remote patient monitoring, diabetic foot ulcer, diabetes, neuropathy, chronic care management, early detection of neuropathic wounds

## Abstract

**Background:**

Diabetic foot ulcers (DFUs) are a leading cause of hospitalizations, amputations, and health care costs among individuals with diabetes, often due to delayed detection and treatment. Early identification of skin changes is critical for preventing ulcer progression, yet daily visual foot self-inspections are often limited by impaired mobility, visual deficits, and poor compliance. Although some foot-focused remote patient monitoring technologies are in use, most of these approaches do not provide standardized, full-color visual documentation, which can affect clinicians’ ability to interpret findings using familiar visual cues.

**Objective:**

This formative evaluation aimed to assess the usability, compliance, and feasibility of an in-home full-color imaging scale for the remote monitoring of patients with DFUs to inform the design of larger prospective studies.

**Methods:**

We conducted a retrospective formative feasibility evaluation analyzing 7 months of preexisting data from adults with a history of DFUs who had enrolled in an outpatient remote monitoring program. During this program, participants were provided with an internet-connected in-home scale that captured high-resolution, full-color scans of the plantar surface of the feet during daily use. Scans were securely transmitted to a HIPAA (Health Insurance Portability and Accountability Act)-compliant web portal for podiatrist review. Usability was assessed through compliance with daily scanning, patient and physician surveys, and descriptive review of clinical workflows.

**Results:**

A total of 21 participants aged 43 to 86 years (median 65, IQR 59-74; mean 65.3, SD 10.6 years) were retrospectively analyzed. Participants demonstrated high compliance and feasibility of home use, with device use on 82.5% (3638/4410) of possible days, a median use of 27 days (IQR 21-30) per 30-day period, and a mean use of 23 (SD 6.4) days per 30-day period. Participants reported high satisfaction and minimal burden associated with daily use, with most rating the device as physically easy to use (median 7.0, IQR 7-7; mean 6.9, SD 0.45), expressing willingness to use it daily (median 7.0, IQR 7-7; mean 6.9, SD 0.31), and indicating trust in the system (median 7.0, IQR 7-7; mean 6.9, SD 0.32) on a 7-point Likert scale (1=worst; 7=best). Clinician usability was supported by podiatrist review of 3295 scans, of which 3220 (97.7%) were rated as usable for clinical assessment. Three representative cases illustrated real-world device use and longitudinal image review, demonstrating the ability to visualize skin changes such as foreign body presence, new ulceration, and early skin breakdown.

**Conclusions:**

This retrospective formative evaluation demonstrates the preliminary usability and feasibility of an in-home, full-color imaging scale for daily remote visual monitoring of diabetic feet in a real-world outpatient setting. High compliance and usability in clinical workflows suggest that this approach may support early identification of foot-related concerns. Future prospective studies are warranted to evaluate the impact of this technology on clinical outcomes, including ulcer progression and health care use.

## Introduction

Foot ulcers pose significant morbidity and mortality, particularly among patients with diabetes mellitus, peripheral neuropathy (whether diabetes induced, chemotherapy induced, idiopathic, alcohol induced, or from other metabolic neurodegenerative causes), and peripheral vascular disease. These conditions contribute to a cascade of complications that affect multiple organ systems, but their impact on the feet is especially devastating [1,2]. When left untreated or undiagnosed, ulcers can progress from superficial lesions to deep infections requiring amputation, often leading to permanent disability and a marked decline in quality of life [3,4]. The burden includes extended hospitalizations, missed work, and loss of independence. The recurrence rate of foot ulcers is high, frequently resulting in repeated visits to wound care clinics, rehospitalizations, and additional amputations, with recurrence after healing estimated at 42% within 1 year and 65% within 5 years [5-7]. The cost of a single major diabetic foot amputation averages US $100,000 [8], largely driven by the high costs of hospitalization [5]. Once amputation occurs, the 5-year mortality rate exceeds 50% in patients with diabetes [1,9]. As such, diabetic foot ulcers (DFUs) significantly strain both patients and health care systems, accounting for over US $40 billion in annual treatment costs in the United States alone [8]. Epidemiologically, diabetes, neuropathy, and peripheral vascular disease remain major global health concerns, with 15% to 25% of patients with diabetes expected to develop a foot ulcer in their lifetime [10].

A key driver of poor outcomes in DFUs is delayed detection. Ulcers often begin as small, unnoticed blisters or calluses that go untreated due to loss of protective sensation, visual impairment, or limited mobility [1,4,11]. Daily self-examinations are crucial, but barriers such as mobility, obesity, poor vision, and noncompliance interfere with effective monitoring [11,12]. Insurance coverage further complicates early detection: Medicare, for example, only permits routine foot care every 61 days [13]. These gaps in surveillance can be clinically consequential for high-risk patients as prior studies of hospitalized individuals with severe diabetic foot complications have demonstrated that progression to major amputation can occur within weeks once advanced disease is present, with median times on the order of one month [14].

Remote patient monitoring (RPM) can be a potential strategy to bridge these surveillance gaps and enable earlier clinical intervention. The Centers for Medicare and Medicaid Services (CMS) has endorsed RPM as a cost-effective approach for managing chronic conditions, including diabetes [13]. Prior investigations have demonstrated that, when implemented as intended, RPM can be an effective tool for early detection and prevention of diabetic foot complications [15-18]. Current RPM technologies for diabetic foot care primarily focus on monitoring associated markers of ulcers, such as blood pressure, glucose levels, foot temperature, and plantar pressure points [19-21]. These methods achieve high sensitivity associated with anomalous measurements, with temperature measurements in particular proving to be very promising but with the main drawback of low specificity [16,20,22-25]. However, these methods have not effectively addressed a critical gap—daily visual foot inspections, which have been established as the standard of care in identifying early signs of ulceration [4,21].

Prior research has demonstrated that visual assessment–based RPM offers clinicians effective routine oversight and the ability to intervene quickly when subtle changes occur, potentially reducing hospitalizations and improving outcomes [21,26]. One study that combined thermometry with visual image scanning showed that podiatrists considered the visual scan data to be useful 90% of the time compared to thermal data, which were only useful 12% of the time [21]. The study enumerated three main reasons why podiatrists strongly preferred the visual images over thermal data: (1) low specificity of temperature-based analyses for DFU prediction, (2) importance of visual images for identifying preulcerative conditions such as calluses and foreign bodies, and (3) pre-emption of inflammation and subsequent temperature increase due to more early clinical referrals triggered by visual images [21].

In contrast to existing remote monitoring technologies that track metrics such as foot temperature [20,22-25], full-color high-resolution remote imaging of the foot offers the additional benefit of providing direct images of the plantar foot area, allowing health care providers to visualize abnormalities remotely, often before more severe symptoms manifest. This approach may offer several potential advantages: (1) images require minimal specialized interpretation compared to temperature gradient analysis, (2) visual documentation provides objective evidence for clinical decision-making and care coordination, and (3) the scanning and review protocol closely mimics standard podiatric foot examination workflows [1,2,11].

Despite these advantages, the adoption of image-based RPM in real-world settings remains limited by usability barriers. Prior studies report that, while both patients and clinicians recognize the potential value of wound imaging, capturing foot images using modalities such as smartphones can be challenging due to limited mobility, the need for caregiver assistance, and inconsistent image quality or sequencing [26]. Additionally, variability in digital literacy may reduce engagement with mobile health tools [26]. These barriers highlight a gap between the clinical promise of visual RPM and its practical usability in high-risk populations.

Recent advances in consumer-grade imaging technology and secure digital health platforms create new opportunities to address these limitations through passive, in-home solutions that minimize user burden and automate image capture during routine use. However, the feasibility, usability, and preliminary clinical utility of such systems in real-world diabetic foot care settings remain underexplored. This study aimed to conduct a formative evaluation of a novel, in-home, full-color foot imaging device among high-risk patients with a history of DFUs, with a focus on usability, patient compliance, and integration into routine clinical workflows.

## Methods

### Study Design and Participant Recruitment

We conducted a retrospective formative feasibility evaluation using deidentified foot imaging and compliance data obtained from a pilot RPM program for high-risk patients with DFUs operated by Empo Health. Records collected between April 2024 and November 2024 from adult patients enrolled in remote monitoring through 3 podiatric clinics in the Baltimore metropolitan area, Maryland, were retrospectively evaluated. This analysis included up to 7 months of RPM data per participant, consisting of a 1-month device break-in period, 3 months of structured daily use with formal compliance tracking, and an additional optional 3-month period of unstructured use.

This study was designed as a formative evaluation to assess device usability, patient compliance, and real-world feasibility; it was not designed or powered to evaluate definitive clinical outcomes such as ulcer incidence, hospitalization rates, or amputation prevention. The dataset included patient demographics, device use dates and daily compliance metrics, transmitted foot scans and their podiatrist-rated clinical usability, time-stamped clinical notes from podiatrist follow-up, and documentation of clinical interventions prompted by image review.

**Figure 1.**
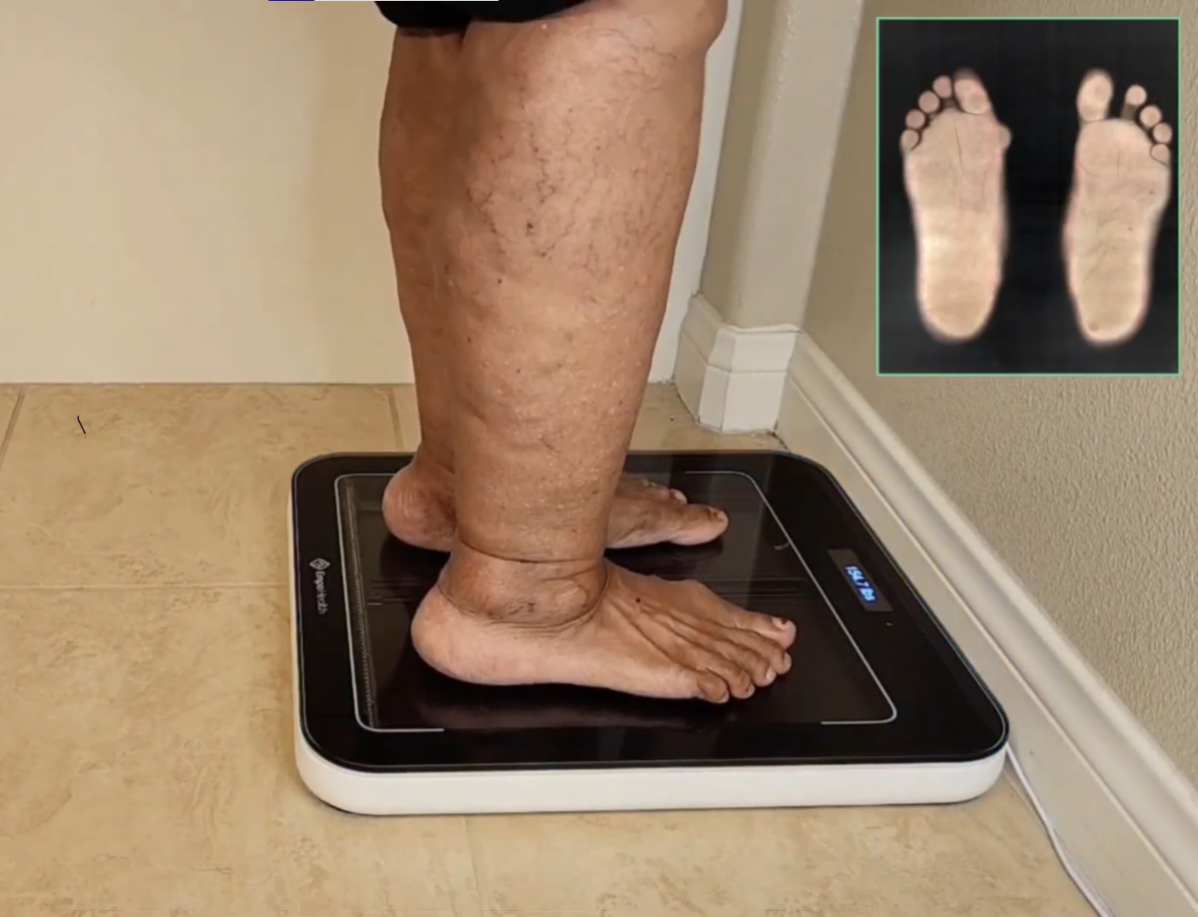
The Empo Footprint with an associated high-resolution, full-color foot scan as uploaded automatically to the Empo Remote Health Link portal.

Monitoring services were provided following podiatrist recommendation and clinical assessment of patient eligibility. Eligibility criteria included a documented history of DFU, high-risk status, and confirmed internet connectivity. Each participant was provided with an internet-connected, full-color imaging scale (Empo Footprint) for in-home use (Figure 1). The device is a full-color, high-resolution, internet-connected foot imaging scale designed for a patient to stand on every day to capture high-resolution scans of the soles of their feet, as well as to measure their weight. These scans are automatically and securely transmitted to health care providers via a HIPAA (Health Insurance Portability and Accountability Act)-compliant web portal for remote clinician data review as part of the monitoring program.

### Data Collection

Participants were monitored for up to 7 months as part of the RPM program from which this retrospective dataset was derived. During the first 4 months, participants were asked to use the scale daily. The first month served as a break-in period, followed by 3 months of formal compliance tracking for usability assessments. The scale was designed to be “senior ready,” requiring no advanced technical skills or device configuration by the user.

Remote visual assessments were conducted asynchronously by the participating podiatrists, who reviewed all transmitted foot scans on a daily basis. Image review was performed by the treating podiatrist for each participant’s site, ensuring clinical familiarity with each patient’s baseline foot condition. Consistent with real-world RPM practices, reviewers were not blinded to participant medical history or prior imaging. No independent adjudication or interrater reliability assessment was performed as this formative evaluation was designed to assess real-world clinical feasibility rather than standardized diagnostic accuracy. Clinical findings identified during image review were corroborated through in-person examination where applicable and documented in participant medical records.

As part of the image review process, podiatrists were asked to complete an optional standardized questionnaire for each foot scan confirming whether (1) the image quality was sufficient for remote foot assessment, (2) there were any changes compared to prior scans, and (3) any additional clinical actions were needed beyond continued monitoring. For any concerns (eg, poor image quality, observed changes, or recommended actions), the clinicians were asked to provide a brief explanation. This feedback was used both for evaluation purposes and to enable over-the-air firmware improvements during the course of the usability period.

Following completion of the initial 4-month usability period, participants were offered the option to continue using the device for an additional 3 months of free, unstructured monitoring. Of the 21 participants, 20 (95.2%) elected to continue device use during this extended period for the fifth month, and 18 (85.7%) elected to continue using the device for the remaining 2 months.

### Data Analysis

Usability was assessed descriptively through participant compliance with daily foot scan capture and podiatrist-rated image usability. Compliance was defined as the proportion of days in which participants successfully completed at least one foot scan relative to the total number of expected monitoring days during the compliance tracking period. The proportion of participant-months meeting or exceeding 16 days of device use per 30-day period was also assessed as a secondary compliance metric. The 16-day threshold was selected based on contemporaneous CMS RPM reimbursement standards, which at the time of the study required a minimum of 16 days of monthly device use to qualify for RPM billing [27]. Although this program was not reimbursed under CMS at the time, this threshold represented the most widely accepted benchmark for clinically meaningful RPM engagement and was therefore adopted as a reference standard for this evaluation. Image usability was defined as the proportion of foot scans deemed sufficient for clinical assessment based on podiatrist responses during image review.

Following completion of device use (up to 7 months per participant), a retrospective analysis was performed using the complete dataset that had been deidentified prior to access by the research team. This analysis incorporated data from both the formal usability period (months 2-4) and the extended monitoring period (months 5-7) to assess longer-term compliance patterns and instances of clinical action associated with image review.

### Ethical Considerations

This retrospective analysis of the complete 7-month dataset was reviewed and determined to be exempt under Title 45 of the Code of Federal Regulations part 46.104(d)(4) by the Advarra Institutional Review Board. The exemption determination was based on the analysis of existing, deidentified data collected during routine clinical care. In accordance with research ethics, all patient information, including foot scans, was deidentified before analysis.

## Results

### Study Population and Characteristics

The retrospective analysis included data from 21 patients aged 43 to 86 years (median 65 years, IQR 59-74; mean 65.3, SD 10.6 years; n=17, 81% male and n=4, 19% female) enrolled in remote monitoring (Table 1).

During the 3-month required use phase of the study, podiatrist remote image review identified 7 unique indicators of early ulcers among 33.3% (7/21) of the participants, leading to clinical actions. Table 2 summarizes all detected findings by type, clinical response, and outcome. These findings included bleeding (n=4), callus color changes prompting in-person evaluation (n=2), foreign body impaction (n=1), and subcutaneous ulcer development (n=1). All identified findings prompted clinical follow-up, ranging from telephone-based guidance to expedited in-person evaluation.

During the subsequent 3-month optional continuation period, compliance remained high, with device use on 82.2% (1554/1890) of possible days. In our findings, we present 3 selected cases (participants 1, 14, and 27) to illustrate the spectrum of findings and clinical responses facilitated by daily remote visual monitoring.

**Table 1.** Demographics and relevant medical history of the 21 participants. Missing IDs indicate those who declined to participate after signing a participation release but prior to receiving the study device^a^.

Participant ID	Age (y)	Sex	Amputation history	Other relevant medical history
1	63	Male	—^b^	—
2	65	Male	—	—
3	68	Male	—	—
4	69	Female	—	—
5	71	Male	Left second toe and metatarsal head amputation	—
7	77	Female	—	—
8	61	Male	Second and fifth toe amputation	—
9	43	Male	Left first and second digit amputation	—
11	49	Male	—	—
13	54	Male	Right third digit amputation	—
14	53	Male	Left first digit amputation	—
15	76	Male	—	—
17	74	Male	—	—
19	71	Male	—	—
20	86	Male	—	—
21	76	Male	—	—
23	60	Female	Left first digit amputation	—
24	75	Male	—	—
25	58	Male	Left fifth digit amputation	—
26	64	Female	Right fifth digit amputation	Legally blind
27	59	Male	—	—

a Median age 65 years; mean age 65.3, SD 10.6 years.

b No applicable medical history.

**Table 2.** Summary of all clinical findings identified through remote image review (n=7 participants).

Participant ID	Finding type	Detection date	Response	Outcome
1	Bleeding from a skin tear	May 18, 2024	Changed the at-home care plan	The podiatrist instructed the patient to soak their feet before removing the bandages to help loosen the adhesive and prevent skin irritation or tearing.
14	Foreign object	May 18, 2024	Called the patient and asked them to step on the device	A new image was uploaded, revealing that the foreign object was not penetrating the skin; the object was removed.
9	Increased drainage and bleeding	June 9, 2024	Scheduled the patient for an in-person visit	The patient was prescribed antibiotics; infection status was monitored and confirmed to be improving at follow-up.
27	Color change in the lateral eschar or callus	June 27, 2024	Scheduled the patient for an in-person visit	Predebridement and postdebridement assessment was performed by the podiatrist; the underlying ulcer was identified during debridement and treated.
17	Color change in the central left subfourth metatarsal head	June 28, 2024	Scheduled the patient for an in-person visit	The podiatrist applied silver nitrate to the patient’s foot; in-person debridement revealed an underlying ulcer beneath the callus.
8	Bleeding from the patient’s right fourth toe	July 14, 2024	Scheduled the patient for an in-person visit	The patient’s traumatic skin tear was treated by the care team.
15	Bleeding on the participant’s left foot	August 6, 2024	Scheduled the patient for an in-person visit	The care team trimmed the callus around the ulcer, applied silver oxide, and covered it with gauze.

### Clinical Utility and Case Illustrations

#### Participant 1: Recurrent Skin Tears Leading to Care Plan Modification

**Figure 2.**
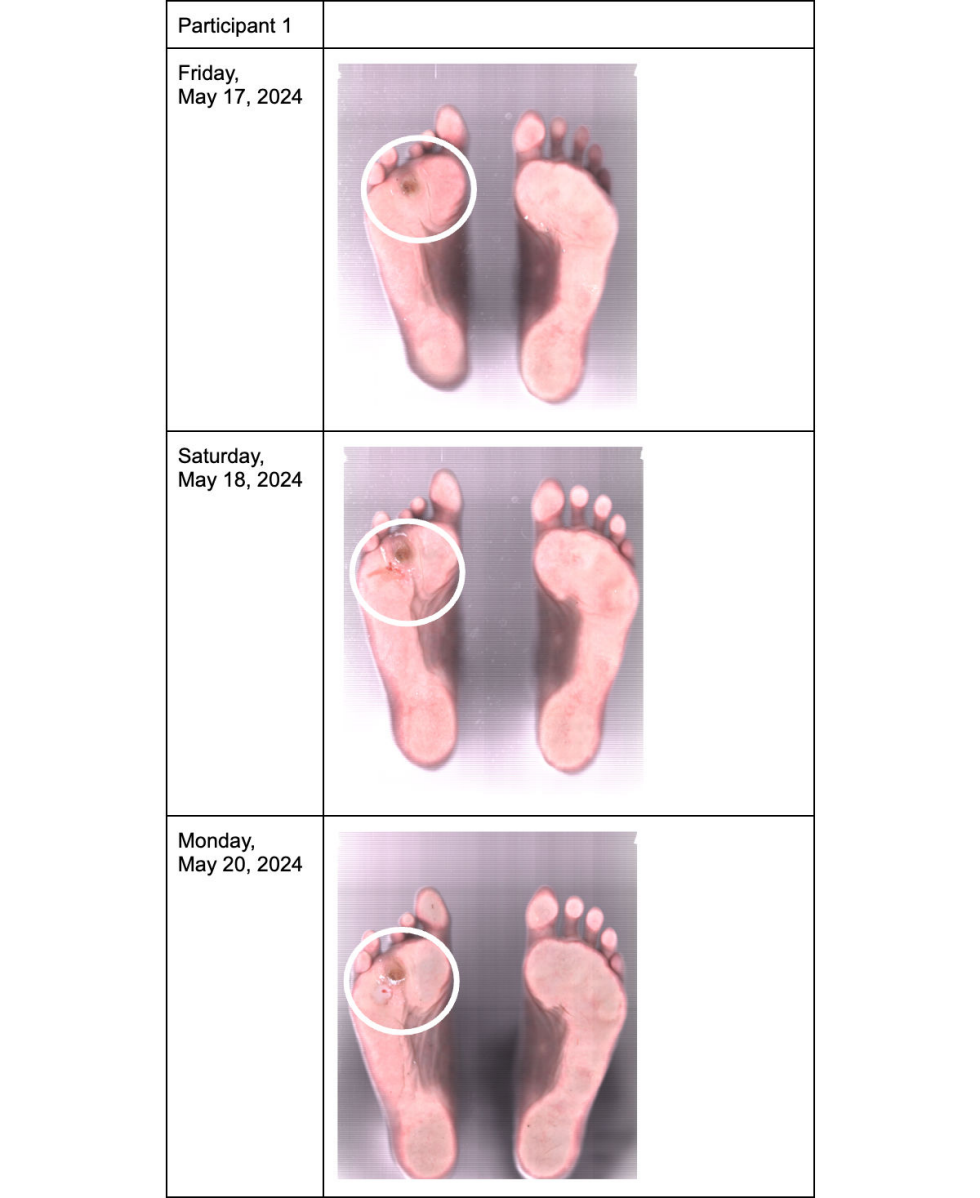
Empo Footprint scans of participant 1. The area of interest has been highlighted using a white circle for demonstration purposes. The first image, captured on May 17, 2024, depicts a preexisting callus on the right foot. The second image, captured on May 18, 2024, shows discoloration consistent with potential bleeding on the patient’s right foot, prompting action by the care team the following business day. The third image, captured on May 20, 2024, depicts the appearance of the wound on the day that the care team reviewed the images and adjusted the care plan.

Participant 1, who had a history of foot ulcers, experienced a sudden overnight wound, which was identified during the routine screening of their remote foot scans via the remote web portal on Saturday, May 18, 2024 (Figure 2). The clinical care team received and reviewed the images the following business day on Monday, May 20, 2024, and discussed the results over a phone call with the participant. The podiatrist then adjusted the treatment to a new bandage method of soaking the foot before removing the bandage to prevent skin tears given the participant’s brittle skin and history of skin breakdowns. The participant was also instructed to add a protective layer beneath the bandage because the adhesive may have been causing skin damage. Over the study period, 2 additional tears in the same area were observed on scans on Friday, June 7, 2024, and Friday, August 9, 2024. After these events, the podiatrist changed the patient’s engagement plan to include phone call reminders to help the participant follow their care plan. In-person visits with the podiatrist were scheduled every 61 days. In summary, real-time scans led to (1) earlier detection of skin tears, (2) a better understanding of the underlying trigger for skin breakdown, and (3) execution of a different engagement plan to include phone call reminders to help the participant follow their care plan.

#### Participant 14: Foreign Body Detection Leading to Removal of Object and Patient Education

**Figure 3.**
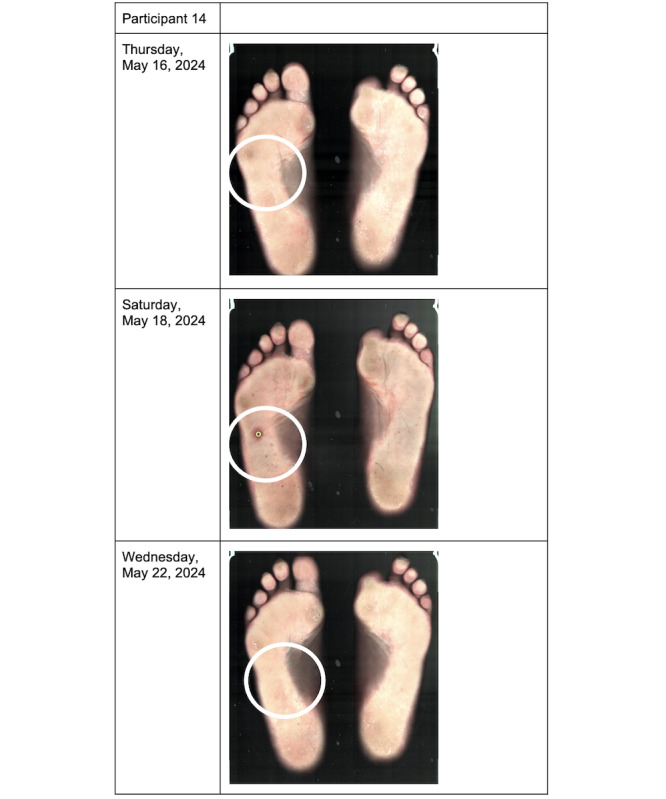
Empo Footprint scans of participant 14. The area of interest has been highlighted using a white circle for demonstration purposes. The baseline scan from May 16, 2024, showed no visible abnormality. A routine remote screening on May 18, 2024, identified a suspected foreign body lodged in the plantar surface of the right foot, prompting action by the care team. A subsequent scan on May 22, 2024, confirmed removal of the foreign body without evidence of skin damage or ulcer formation. This case illustrates how remote patient monitoring enabled early detection, timely intervention, prevention of tissue injury, and avoidance of an in-person clinic visit.

Participant 14, who had previously undergone a toe amputation, had a foreign body lodged in their right foot, which was identified via the routine screening of their remote foot scans captured on May 18, 2024 (Figure 3). The podiatrist noted that, while the foreign object was detected before it could penetrate the skin, it still posed a risk of pressure damage and skin breakdown. During a phone call triggered by the review of this image, the participant reported that they were unaware of the object’s presence because they did not feel any discomfort in their feet. This served as an educational opportunity for the care team to convey the importance of refraining from walking around their home barefoot. A follow-up screening demonstrated that the object had been removed safely from the foot before any lasting skin damage had occurred. This reassuring scan prevented an in-person follow-up in the clinic to ensure a lack of skin damage. In this case, RPM led to both the detection of an impacted foreign object in the foot within 2 days of original insult and the prevention of an in-person office visit and associated economic costs as RPM provided evidence of lack of skin damage.

#### Participant 27: Early Detection of Subcutaneous Ulcer Development Leading to In-Clinic Intervention

**Figure 4.**
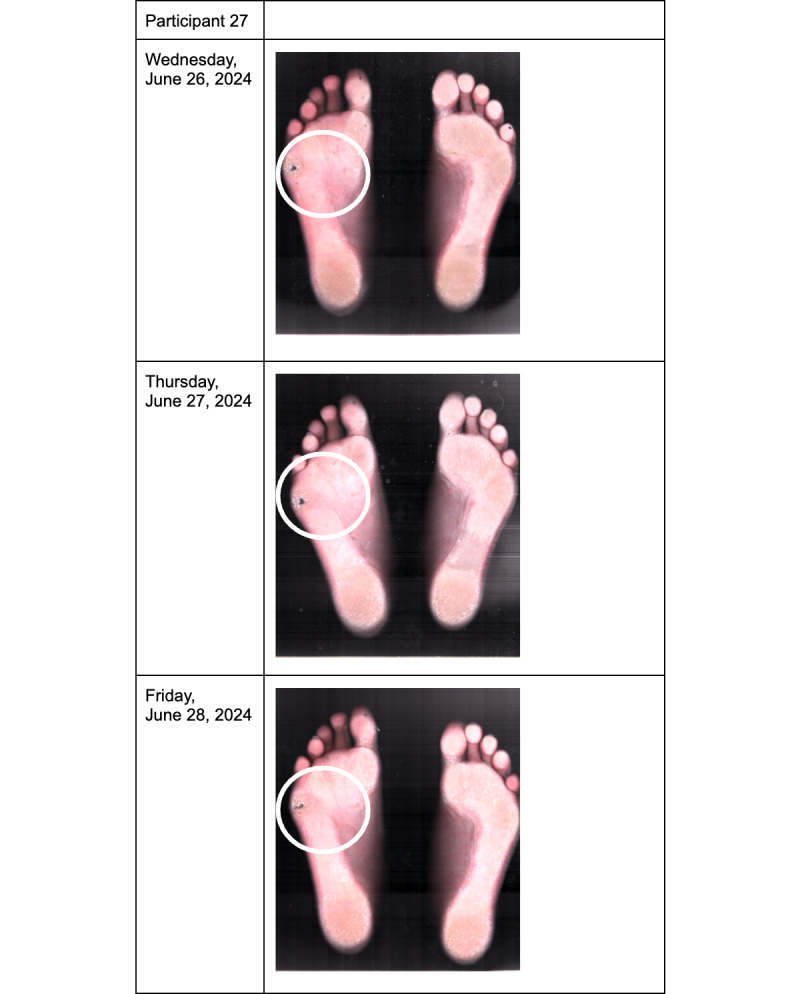
Empo Footprint scans of participant 27. The area of interest has been highlighted using a white circle for demonstration purposes. The first image, captured on June 26, 2024, depicts one day before a change in the right foot was identified on June 27, 2024. The third image was captured on June 28, 2024, one day after the change was detected. At a scheduled clinic visit on July 1, 2024, examination confirmed a dissecting intracutaneous hematoma with underlying ulceration.

Participant 27 had a color change indicating early development of a subcutaneous ulcer identified through full-color remote imaging on June 27, 2024 (Figure 4). At a prescheduled clinic visit on July 1, 2024, the podiatrist confirmed ulcer development beneath the callus, and treatment was initiated. Upon in-person examination, it was found that there was a dissecting intracutaneous hematoma on the side of the foot, leading to a purple discoloration. The podiatrist performed a debridement, discovering and treating a wound hidden beneath the callus. In this case, RPM detected an ulcer before presentation in the clinic and led to earlier intervention.

### Compliance Data

**Figure 5.**
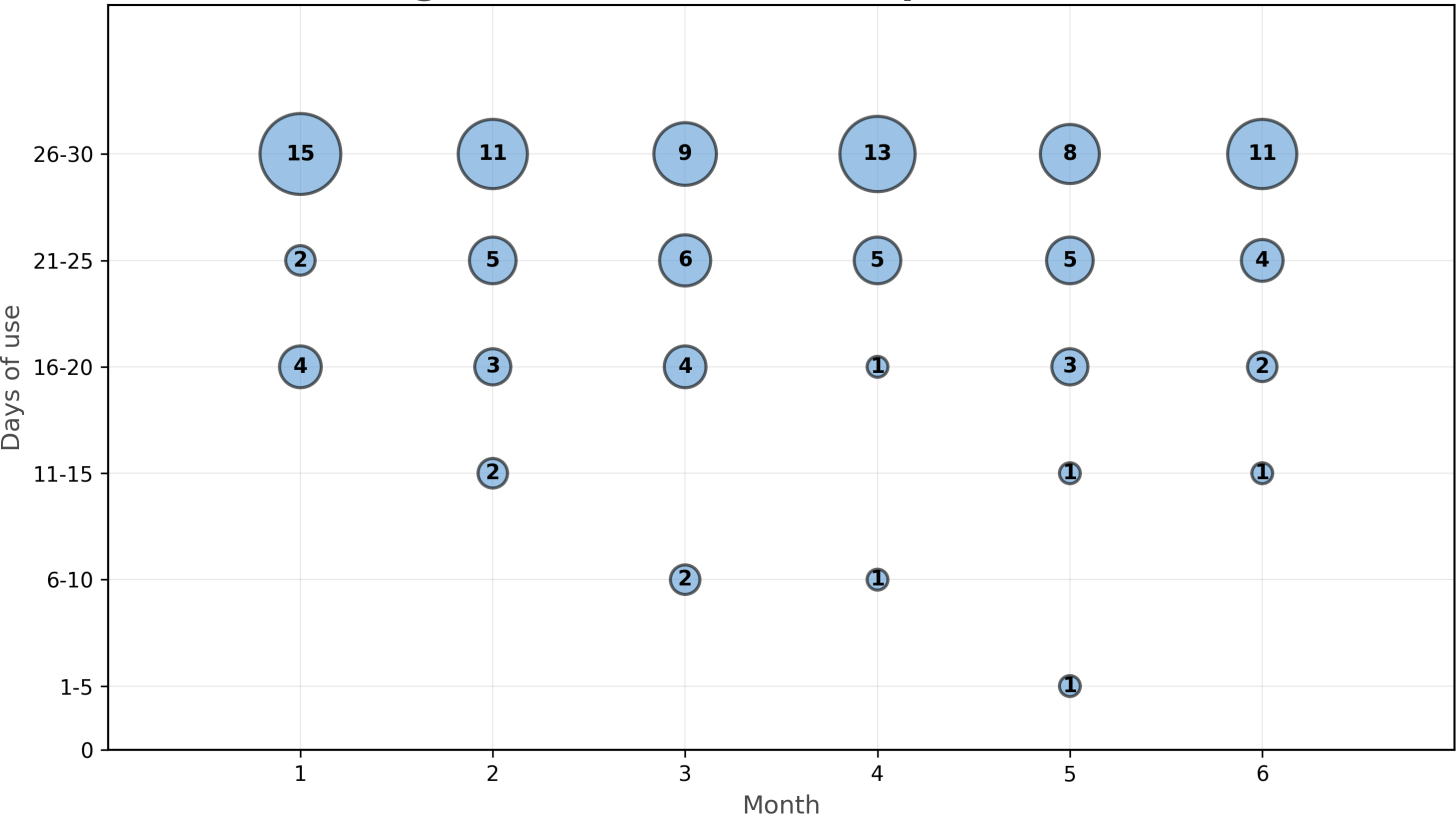
Days of device use per participant by month. Each bubble shows the number of participants (displayed inside) whose device use fell within each 5-day range. Bubble size is proportional to participant count.

Compliance was assessed using rolling 30-day participant-months, with 3 participant-months analyzed per participant (N=21) following the initial break-in period. The 21 participants as a population adhered to near-daily use of their in-home foot monitoring scales, as shown in Figure 5. Across the study period, participants used the device on 82.5% (3638/4410) of all possible days. Median device use was 27 out of 30 days per participant-month, and mean use was 23 out of 30 days over the 6-month study period.

A total of 88% (111/126) of the 30-day participant-months met or exceeded the target device use of 16 days for 6 months (corresponding to a device use of more than half of the days in each month). Of the 7 instances in which participants did not meet the 16-day monthly use target, 3 reflected near-threshold compliance, with device use of 14 to 15 days. These metrics included any time participants were unavailable to step on their device due to travel, vacation, or other medical issues.

### User Experience Survey

After 4 months of participation, 95.2% (20/21) of the participants completed a user experience survey in which they reported high levels of satisfaction with and ease of use of the remote foot monitoring scale. In total, 4.8% (1/21) of the participants were unable to complete the survey due to an unrelated hospitalization. Using a 7-point Likert scale where 1 represented the worst score and 7 represented the best score, most participants reported that they found the device physically easy to use (median 7.0, IQR 7-7; mean 6.9, SD 0.45), were willing to use it daily (median 7.0, IQR 7-7; mean 6.9, SD 0.31), and trusted the system’s ability to detect foot-related issues (median 7.0, IQR 7-7; mean 6.9, SD 0.32). Table 3 summarizes the survey results.

**Table 3.** Participant experience survey responses for 20 participants after 4 months of using the Empo Footprint, rated on a 7-point Likert scale where 1 represented “strongly disagree” and 7 represented “strongly agree.”

Survey question	Score, mean (SD)
What has been your level of willingness to use the scale daily?	6.9 (0.31)
Physical ease of use—I can get on and off the scale very easily.	6.9 (0.45)
I trust the ability of the system to detect foot issues (ulcers and/or related health problem).	6.9 (0.32)

### Summary of Missed Device Use Days

All missed device use days recorded during the study period are summarized in Table 4. Missed days were categorized based on whether a reason for nonuse was communicated to the study team in advance (expected) or occurred without prior notification (unexpected). Expected missed use days included participant-reported travel or medical incidents that prevented device use. Unexpected missed use days included travel or medical incidents that were identified after the missed day occurred and were not communicated to the study team in advance.

Missed use days for which no reason was documented were categorized as “no reason documented.” No missed use days were attributed to device failures or technical malfunctions during the study period. Transmission errors leading to missing images were not classified as missed device use days as participants used the device and weight data were captured; these events reflected incomplete data capture (missing image) rather than nonuse or device failure.

**Table 4.** Summary of missed device use across all participants throughout the 6-month study period. Expected missed days were reported to the study team in advance; unexpected missed days were identified retrospectively. Transmission errors resulting in missing images but with confirmed device use were not classified as missed use days.

Reason for nonuse	Missed days (n=810), n (%)	Proportion of total days (n=4229), n (%)
Travel (unexpected+expected)	154 (19)	154 (3.6)
Medical incident (unexpected+expected)	142 (17.5)	142 (3.4)
No reason documented	514 (63.5)	514 (12.2)
Device failure	0 (0)	0 (0)

### Image Usability

During the study period, podiatrists completed surveys for 3295 total foot scans and identified 3220 (97.7%) as usable (Table 5). The 2.3% (n=75) of the scans that were unusable were primarily attributed to patient-related factors or image quality issues, such as patients wearing socks or shoes or movement during the foot scan (n=53, 1.6%), and technical instances in which a patient stepped on the device but the scanning mechanism did not capture an image, resulting in only weight data being recorded (n=22, 0.7%).

**Table 5.** Total image usability throughout the study as assessed by treating podiatrists (n=3295 total screenings reviewed)*.*

Metric	Frequency, n (%)
Accepted (usable images)	3220 (97.7)
Rejected (unusable images)	75 (2.3)
Reason: socks or shoes worn or foot blurred or obstructed	53 (1.6)
Reason: missing image (partial transmission or only weight captured)	22 (0.7)

### Device Usability

Device performance was monitored throughout the study period. No hardware failures, safety incidents, or data security breaches were reported. Transmission failures were uncommon, observed in just 0.7% (22/3295) of instances. In these cases, the system recorded only weight data without capturing a foot scan, likely due to brief connectivity interruptions or improper user positioning. These events were captured in the image usability analysis and did not result in any gap in participant monitoring as daily scanning continued uninterrupted. No device replacements were required during the study period.

## Discussion

### Principal Findings

This retrospective formative study highlights the usability and real-world feasibility of daily remote foot monitoring via full-color images and its potential role in supporting early clinical assessment of foot changes in a high-risk diabetic foot population. Across 21 participants over 7 months, the device achieved an overall compliance rate of 82.5% (3638/4410; median 27/30, IQR 21-30 days per month), with 97.7% (3220/3295) of submitted images rated as clinically acceptable for remote assessment. Patient-reported usability ratings were consistently high, with participants reporting ease of use and willingness to continue daily monitoring. Notably, compliance remained high despite the study being conducted during the summer months, a period typically associated with disruptions to daily health routines. Podiatrists identified 7 clinically concerning findings across 7 unique participants, all of which prompted clinical follow-up ranging from telephone-based guidance to expedited in-person evaluation. These aggregate findings collectively support the feasibility and acceptability of integrating this RPM modality into routine care for high-risk patients.

Prior to the RPM program enrollment, podiatrists reported that these participants were typically noncompliant with performing their recommended diabetic foot care routines, including routine visual foot inspections, which contributed to delayed identification of skin changes and a history of ulceration and, in some cases, amputation. By enabling high-quality daily visual inspections, the scale-provided foot scans facilitated earlier detection of skin changes that may have otherwise progressed without timely intervention.

Three illustrative cases demonstrate the range of clinical scenarios in which daily visual monitoring facilitated timely clinical response: (1) enabling detection of acute changes (foreign body and skin tears) within 24 to 48 hours rather than at the next scheduled visit (typically 61 days apart under Medicare guidelines), (2) facilitating asynchronous review to distinguish findings requiring urgent in-person evaluation from those manageable through telehealth guidance, and (3) providing serial documentation to identify patterns (eg, recurrent skin breakdown) that may warrant care protocol modifications. However, as a single-arm observational study, these findings represent preliminary observations of feasibility rather than evidence of clinical effectiveness, which would require controlled comparison.

These cases also highlight the limitations of traditional periodic in-office examinations and reliance on in-home unassisted patient self-inspections. Foot ulcers more commonly develop in individuals with physical and sensory limitations, including impaired vision, obesity, limited mobility, and peripheral neuropathy [1]. Thus, unassisted patient self-inspections are often unreliable [26,28], as demonstrated in our case series: participant 14 was unaware of the foreign object in their foot, whereas participant 27 would not have appreciated the subtle changes in callus color. Additionally, poor access to care and communication with health care providers in between in-person visits also leave substantial gaps in care [1]. In our case series, participants were typically scheduled for in-office visits every 61 days, meaning that injuries detected via RPM may not have otherwise been identified until several weeks later. Daily visual monitoring provided by the imaging scale may help address several critical limitations of existing approaches by enabling visualization of acute changes within 24 to 48 hours rather than weeks between clinic visits, facilitating asynchronous clinical review and supporting bidirectional communication between patients and health care providers.

### Comparison With Prior Work

Acknowledging the inherent limitations of a single-arm, sponsor-funded feasibility study, the compliance rate of 82.5% (3638/4410) overall (median 27/30 days per month) in this study nonetheless compares favorably to that of prior RPM studies in chronic disease management, which have reported compliance rates ranging from 45.9% to 82.8% [20-23,29]. This level of engagement in our study was supported by strong patient ratings for ease of use and willingness to continue using the device. These data suggest the acceptability of integrating an RPM device into daily routines even for older adults or those with mobility challenges, as in our case series. Notably, this study was conducted during the summer months, a period typically associated with lower compliance to prescribed care routines due to travel, vacations, and disruptions to daily schedules [30,31]. Despite this, participants demonstrated high compliance with daily monitoring.

The clinical acceptance rate of 97.7% (3220/3295) of scanned foot images in this study was also encouraging and compares well to a prior diabetic foot surveillance study that used mobile phone images, where clinical adequacies (adequacy of images collected via self-photo, selfie stick, and another party) were 74.1%, 83.7%, and 92.6%, respectively [12].

### Implications for Clinical Practice and Future Research

The findings from this formative evaluation suggest that full-color image–based RPM may be a feasible and usable approach for supporting remote foot surveillance in high-risk patients. By enabling consistent, home-based visual assessment of the plantar surface, this modality addresses a known gap in existing RPM strategies, which have largely focused on indirect physiological measures rather than direct visualization of skin integrity. Prior studies of RPM interventions in diabetic foot care have demonstrated that earlier identification of concerning changes and timely clinical response may reduce ulcer progression and hospital admissions, with substantial cost savings [25,32]. Moreover, effective remote foot monitoring may contribute to maintaining patients’ mobility and independence, improving their quality of life, and reducing the risk of emotional and psychological impacts associated with chronic foot ulcers and amputation.

In this context, full-color image–based RPM may offer a complementary approach by facilitating remote clinical assessment of early skin changes that might otherwise go undetected. Although this study was not designed to evaluate clinical outcomes, the observed adherence, image reliability, and illustrative cases of early clinical action suggest that this approach warrants further prospective investigation. Future studies should evaluate whether integration of daily visual monitoring into routine care can improve clinical outcomes, reduce health care use, and enhance patient quality of life.

### Limitations

This study has several limitations. As a retrospective formative feasibility evaluation, it provides descriptive insights into the use of RPM in a small patient population. The relatively small sample size and lack of sufficient gender and racial diversity limit the generalizability of the results. The absence of a control group also limits the ability to directly compare outcomes and determine the full impact of the device. This study was conducted over 210 days, with the first 30 days being the break-in period for device use, and longer studies are necessary to confirm outcomes and demonstrate long-term usability. Although the compliance rates were high for this relatively short study, longer follow-up periods are also crucial to assess sustained compliance, particularly in less motivated patients. Additionally, most missed use days (514/810, 63.5%) had no documented reason, which is consistent with real-world RPM programs where participants are not required to report nonuse, but this limits the ability to distinguish device-related barriers from lifestyle factors.

Another limitation of the study design is that image review was performed by treating podiatrists who were not blinded to participant history, reflecting real-world RPM workflows in which clinical assessments are made with access to patient context. However, this approach introduces the potential for confirmation bias in clinical assessments. The absence of independent review and interrater reliability measurement limits the reproducibility of the findings. Future studies should include larger and more diverse populations and use more rigorous controlled study designs. Blinded, independent image adjudication and formal interrater reliability assessment should also be incorporated to enhance the validity, reproducibility, and generalizability of the clinical findings.

### Conclusions

Prior studies have demonstrated that RPM for diabetic foot care may support earlier identification and management of skin changes. Daily screening of foot scans may enable health care providers to identify and address subtle changes in foot health before they escalate into more serious complications such as infections, hospitalizations, or amputations. In this study, high adherence, clinical image usability, and illustrative cases of early clinical action suggest that this approach is feasible and acceptable for home use. However, as this study was not designed to evaluate clinical outcomes, these findings should be interpreted as preliminary. Given the financial relationships between the study sponsors and most contributing authors, these preliminary findings should be interpreted with caution and are best viewed as hypothesis generating. Independent replication by investigators without financial ties to the technology is a critical next step. Further prospective studies are needed to determine whether integration of daily visual monitoring into routine care can improve clinical outcomes, reduce health care use, and enhance patient quality of life.

## Abbreviations

CMS: Centers for Medicare and Medicaid Services
DFU: diabetic foot ulcer
HIPAA: Health Insurance Portability and Accountability Act
RPM: remote patient monitoring
